# Evaluating the relationship of blood pressure, plasma angiotensin peptides and aldosterone with cognitive functions in patients with hypertension

**DOI:** 10.17179/excli2016-725

**Published:** 2017-03-10

**Authors:** Selçuk Sen, Nermin Gürel, Baran Ufuktepe, Zeynep Günes Özünal, Çagla Büyüklü, Yagiz Üresin

**Affiliations:** 1 Department of Medical Pharmacology, Istanbul Faculty of Medicine, Istanbul University, 34390 Fatih, Istanbul, Turkey; 2 Department of Neuroscience, Institute of Experimental Medicine, Istanbul University, 34393 Fatih, Istanbul, Turkey

**Keywords:** hypertension, cognitive functions, angiotensin, aldosterone

## Abstract

Renin Angiotensin Aldosterone System (RAAS) plays an important role in the development of hypertension. On the other hand, hypertension is a well-known and independent risk factor for cognitive impairment. The aim of the present study was to evaluate the relationship of blood pressure control, plasma angiotensin peptides and aldosterone with cognitive functions. Forty-one patients who were under treatment with the same antihypertensive medications for at least three months were included in the study. Plasma angiotensin II, angiotensin 1-7, angiotensin IV, and aldosterone concentrations were analyzed using an enzyme-linked immunosorbent assay (ELISA). Standardized Mini Mental State Examination (SMMSE) was used to evaluate cognitive functions. When the participants were grouped according to their SMMSE scores (cut-off value: 26 points), we determined significant differences between systolic blood pressure (SBP) levels, diastolic blood pressure levels, plasma angiotensin II and angiotensin 1-7 concentrations of the groups. When the participants were stratified according to their SBP levels (cut-off value: 140 mm Hg), we found significant differences in SMMSE scores and plasma angiotensin IV concentrations of the groups. A negative correlation between SBP and SMMSE scores and strong linear correlations among angiotensin peptides levels were determined. The relationship found between SBP and SMMSE in the present study was compatible with the literature. Our 33 patients were using at least one angiotensin II receptor blocker (ARB). Regarding AT1 receptor blockage, the significant association between higher SMMSE scores and increased angiotensin peptides may support a finding that ARBs prevent dementia and improve cognitive function. Further larger studies are needed to confirm and prove the relation of RAAS biochemical parameters with cognitive function.

## Introduction

The prevalence of hypertension is correlated with age, seen in nearly 50 % of individuals aged between 60-69 years and affects ¾ of people aged more than 70 years (Burt et al., 1995[7]). With the increased awareness of hypertension over the last 30-40 years, there has been a significant decrease in deaths due to cerebrovascular and cardiovascular diseases. These data seen in the literature confirm that hypertension is an independent risk factor in the development of cerebrovascular and cardiovascular diseases. 

**Figure 1:**
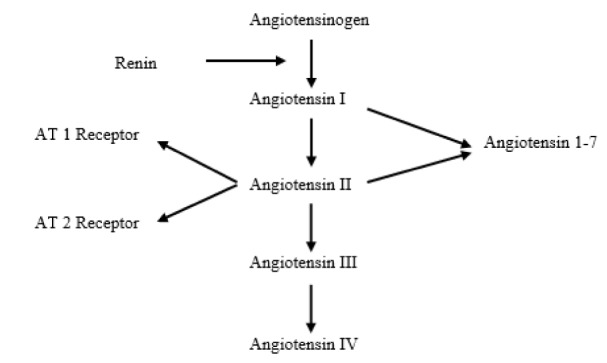
The renin-angiotensin-aldosterone system

The renin angiotensin aldosterone system (RAAS) plays an important role in the development of hypertension. RAAS, being an important mechanism in electrolyte balance and blood pressure regulation, also leads to cardiac and renal changes via neuronal, hormonal, and metabolic effects. For this reason, RAAS blockage is very important for the prevention of hypertension and its complications. RAAS is a complicated system with several parameters (Figure 1(Fig. 1)). Each day we acquaint ourselves with new insights into the role of RAAS in the pathophysiology of cardiovascular disorders (Tusukomato and Kitakaze, 2013[31]).

The relation between cognitive function and hypertension has become a leading area of interest in recent years. Independent from blood pressure and blood pressure control, many studies and reviews have investigated the role of RAAS in cognitive function. RAAS-blocking antihypertensive drugs affects this complex system from different points and thus leads to increased or decreased production of different metabolites. Due to their effects on RAAS system, the possible effects of antihypertensive drugs on RAAS parameters are listed in Table 1(Tab. 1). 

Angiotensin II is the major active mediator of RAAS system and most of its effects are provided via angiotensin type I (AT1) and type II (AT2) receptors. Effects such as vasoconstriction, aldosterone release, cardiovascular hypertrophy and hyperplasia, catecholamine release, and tubular sodium retention is provided via AT1 receptor. In addition, AT1 is responsible in the pathogenesis of atherosclerosis and cardiovascular remodeling. In general, the AT2 receptor shows antagonistic effect when compared with the AT1 receptor (Berry et al., 2001[4]). The major effects of angiotensin II via the AT2 receptor are apoptosis, osmoregulation, cerebral blood flow regulation, prostaglandin metabolism, angiogenesis, nitric oxide release, and vasodilatation (Inagami and Senbonmatsu, 2001[21]).

Angiotensin 1-7 is the most pleiotropic bioactive substance of the RAAS system. Angiotensin 1-7 shows its effect via Mas receptors, which are coded by Mas proto-oncogenes and bound to G proteins. The Mas receptor was shown to be a physiologic antagonist to angiotensin II's effect over AT1 (Kostenis et al., 2005[23]). It has been shown in some studies that the underlying reason for the cognitive improvements provided by ACE inhibitors might be related with increased angiotensin 1-7 levels (Tom et al., 2003[30]; Von Bohlen und Halbach and Albrecht, 2006[32]).

Angiotensin IV is a metabolite of angiotensin II. Other than angiotensin II receptor antagonists (ARB), RAAS-blocking agents (ACEi, beta blockers, direct renin inhibitors) also block formation of angiotensin II. For this reason, RAAS blockers (excluding ARB) might also decrease angiotensin IV formation. The positive effects of angiotensin IV on cognitive processes such as learning and memory have been shown in animal studies (Gard, 2008[12]). Although it is yet unclear, increased angiotensin IV levels may be one of the underlying reasons for cognitive improvement provided with ARB use. 

The final metabolite of RAAS, aldosterone, is stimulated by angiotensin II via the AT1 receptor. Aldosterone is known to have a role in the development of cardiovascular diseases such as hypertension, left ventricular hypertrophy, heart failure, and renal failure (Murin, 2005[27])

Cognitive functions can be described as learning, memory, planning, organization, problem solving, focusing and sustaining attention, understanding the environment, and calculation, which reflect the high processing capacity of the brain. Hypertension is one of the early onset cardiovascular diseases. For this reason, hypertension is a good model to investigate the effects of systemic and cardiovascular diseases on cognitive functions (National Research Council (US) Committee on Future Directions for Cognitive Research on Aging, 2000[28]). For evaluating the cognitive functions, we used standardized mini mental test (SMMSE) which is a scale for estimating the severity of cognitive impairment and monitoring the progress. 

In this study, we aimed to investigate the relation of plasma angiotensin II, angiotensin 1-7, angiotensin IV, aldosterone concentrations, and blood pressure with cognitive functions. 

## Methods

### Study population

Forty-one patients with hypertension who were under treatment with the same antihypertensive medications for at least three months and presented to Istanbul Medical Faculty Department of Clinical Pharmacology Polyclinic were included in this cross-sectional study. The study was approved by Istanbul Medical Faculty Clinical Research Ethics Committee and enrolled participants gave their written informed consent. The inclusion and exclusion criteria are listed at Table 2(Tab. 2). We also evaluated the laboratory test results of the patients from the last one month.

### Blood pressure measurement

Blood pressure measurements were done after resting for a minimum of 5 minutes in the sitting position, measured using a clinical-trial-use approved, validated, and calibrated oscillatory blood pressure measurement device (Omron 7051T, Kyoto, Japan). The patients did not take caffeine-containing drinks or smoke cigarettes 30 minutes before measurement. Three measurements were taken with 5-minute intervals and blood pressure level was recorded as the average of those 3 measurements. We used a systolic blood pressure level of 140 mmHg as a cut-off point.

### Biochemical analysis

Plasma specimens were collected to measure plasma angiotensin II, angiotensin IV, angiotensin 1-7, and aldosterone levels using an enzyme-linked immunosorbent assay (ELISA) (Aldosterone ELISA kit - DRG international USA; Human Angiotensin 1-7 ELISA KIT, Human Angiotensin II ELISA KIT, and Human Angiotensin 4 ELISA kit- Eastbiopharm Co. Ltd Hangzhou, China). All specimens were collected between 08:00 and 10:00 a.m.

Patients stayed for 30 minutes in the supine position before their blood was drawn. Venous blood samples were collected in 4 mL tubes with EDTA for plasma aldosterone level measurement. Samples were immediately centrifuged at 1000 rpm for 15 minutes. Venous blood samples for angiotensin peptides were collected in precooled tubes with EDTA then immediately centrifuged at 2000 rpm for 20 minutes at +4 °C and plasma was directly transported into precooled Eppendorf tubes. 

All samples were stored at -80 °C until required for analysis. All samples were analyzed in duplicate.

### Evaluation of cognitive functions

In accordance with the Diagnostic and Statistical Manual of Mental Disorders (4^th ^ed., text rev.; DSM-IV-TR; American Psychiatric Association, 2000) criteria (American Psychiatric Association, 2000[3]), all patients were first interviewed to exclude dementia. We used a standardized mini mental test (SMMSE) in Turkish, which was validated in 2002 by Güngen et al. (2002[15]) to evaluate cognitive function. Mental scores were evaluated over 30 points. We used an SMMSE score of 26 points as a cut-off point. 

### Statistical analysis

Statistical analyses were performed using IBM's Statistical Package for the Social Sciences (SPSS) for Windows version 21.0. The distribution of variables was tested using the Shapiro-Wilk test. For variables with normal distribution, t-test and Pearson's correlation analysis were performed. For variables without normal distribution, Mann-Whitney U test and Spearman's rho correlation analysis were conducted. The results were considered significant if p < 0.05. The results of the parametric tests are presented as mean ± standard deviation and nonparametric tests are presented as median (25^th^ -75^th^ percentiles).

## Results

Forty-one patients (26 women/15 men, mean age 57.41 years (range, 41-69 years) were consecutively enrolled in the study (see Supplementary data). The SMMSE scores of the patients resulted with a mean of 27.37 ± 2.426. Ten patients with a SMMSE score of 26 or lower were considered to have cognitive impairment. Twenty patients with SBP levels of 140 mmHg or higher were considered to have uncontrolled hypertension. The antihypertensive medications of the patients are summarized at Table 3(Tab. 3).

We stratified the patients into two groups according to their SMMSE score and SBP level. The basic characteristics of the study groups were similar and there were no significant differences. The mean age and years since first diagnosis of hypertension of patients with SMMSE scores of 26 or lower were 56.1 ± 5.60 and 8.7 ± 7.66 years, respectively. The mean age and years since first diagnosis of hypertension of patients with SMMSE scores of 27 or higher were 57.84 ± 8.36 and 9.01 ± 6.75 years, respectively. 

The mean age and years since first diagnosis of hypertension of patients with SBP levels of 140 mm Hg or higher were 58.45 ± 7.37 and 9.53 ± 7.06 years, respectively. The mean age and years since first diagnosis of hypertension of patients with SBP levels lower than 140 mm Hg were 56.43 ± 8.15 and 8.37 ± 6.85 years, respectively. 

When participants were grouped as SMMSE score > 26 points (n=31) and SMMSE score ≤ 26 points (n=10), there were significant differences between systolic blood pressure (SBP) levels (p < 0.05), diastolic blood pressure (DBP) levels (p < 0.01), plasma angiotensin II (p < 0.05), and angiotensin 1-7 (p < 0.05) concentrations of the groups, whereas no significant relationship was detected with plasma angiotensin IV and aldosterone concentrations (Table 4(Tab. 4)).

We stratified the patients according to their SBP levels. When participants were grouped as SBP ≥ 140 mm Hg (n=20) and SBP < 140 mm Hg (n=21), the differences of SMMSE scores (p < 0.05) and plasma angiotensin IV concentrations (p < 0.05) were found significant, whereas no significant relationship was found with plasma angiotensin II, angiotensin 1-7, and aldosterone concentrations (Table 5(Tab. 5)). 

A negative correlation between SBP and SMMSE scores (r = -0.367, p = 0.018), negative correlations between plasma angiotensin IV concentration and SBP (r = -0.385, p = 0.013), and plasma angiotensin IV concentration and DBP (r = -0.346, p = 0.027) were detected using correlation analysis. Besides, strong linear correlations among angiotensin peptides levels were determined. The correlations found in the present study are given in Table 6(Tab. 6). There was no correlation between plasma aldosterone levels and any of the study parameters. 

## Discussion

The average life span is increasing worldwide and dementia and cognitive impairment incidence is rising in parallel with the aging population. It is evident that high blood pressure levels are related with cognitive impairment, stroke, and cerebrovascular diseases. Research on the impact of RAAS, which plays a critical role in the development of hypertension, is a leading topic in current clinical trials. Although several articles have investigated the relation between RAAS and cognitive functions, it should be further investigated.

In our study, patients with high systolic blood pressure resulted with low SMMSE scores. The negative correlation between systolic blood pressure and SMMSE shows that hypertension is an independent risk factor in cognitive impairment. In addition, when patients were classified as SMMSE = 26 and > 26, the difference between the diastolic blood pressure levels of the two groups was significant (p < 0.005). The importance of keeping blood pressure levels under control seemed to be important in maintaining cognitive functions in a trial of the elder population (Waldstein and Katzel, 2004[33]).

In research published in 2011, high plasma aldosterone levels were shown to be associated with cognitive impairment. When their results were evaluated in a multiple regression analysis, age (p < 0.001), plasma aldosterone concentration (p < 0.001), and cerebral infarction history (p < 0.005) were reversely associated with SMMSE scores. Mineralo-corticoid receptor blockage was associated with cognitive impairment prevention as well as cardiovascular mortality prevention (Yagi et al., 2011[34]). In our study, there was no correlation between plasma aldosterone concentration and MMSE scores, which may have been related with the trial’s design, study population and the number of patients.

Plasma aldosterone and angiotensin concentrations might be affected by several factors such as age, drugs, Na intake, and posture of the patient. In our study, patients lied down for 30 minutes before their samples were taken and they were also informed about Na diet. Evaluating sensitive parameters such as plasma aldosterone concentrations may be required with high numbers of samples. Besides, increased aldosterone levels were shown to be related with cerebrovascular function and intensive antihypertensive treatment may benefit the most for the patients with high aldosterone levels (Hajjar et al., 2015[17]). As a result, prospective studies with larger sample sizes are needed to confirm the role of aldosterone on cognition. 

Hypertension is related with RAAS activation and endothelial dysfunction. Therefore, it can be associated with abnormal regulation of cerebral blood flow and cognitive impairment (Hajjar et al., 2009[18]). The role of the endothelium in cognitive function, ageing, and hypertension has been highlighted (Dal-Ros et al., 2009[9]; Dimitropoulou et al., 2006[10]; Haberl et al., 1991[16]; Hajjar et al., 2009[18]; Khalil et al., 2007[22]). Angiotensin II, especially via AT1 receptor, impairs endothelial function and plays a pathophysiologic role (Ai et al., 2007[1]; Dal-Ros et al., 2009[9]; Dimitropoulou et al., 2006[10]; Haberl et al., 1991[16]; Hajjar et al., 2009[18]) Although no significant correlation was shown between angiotensin II levels and SMMSE scores, when the SMMSE score of 26 was accepted as a threshold, the difference between the groups was significant (p < 0.05). In the SMMSE > 26 group, angiotensin II levels were also high. Considering the negative effects created by angiotensin II via the AT1 receptor, this could be related to most patients’ (n = 33) ARB use. It is known that angiotensin II has reverse effects via AT1 and AT2 receptors. AT1 receptor blockage by ARBs increases angiotensin II levels and this increased angiotensin II binds to AT2 and other receptors, or converted to other elements of the RAAS system. It is not surprising to observe that positive SMMSE score results in patients using ARB with increased angiotensin II levels. On the other hand, mid-products of RAAS that have positive effects on cognitive function increase. Angiotensin II is also a precursor of several mid-products such as the neuroactive angiotensin IV (Braszko, 2006[5]). In other respects, a hypothesized mechanism suggested that acetylcholine release may be inhibited by angiotensin II and stimulation of AT2 receptor can be a neuroprotective factor (Gorelick and Nyenhuis, 2012[14]).

There was no correlation between angiotensin 1-7 and SMMSE scores. However, when patients were grouped with the SMMSE 26-point threshold, the difference between groups was significant (p < 0.05). Angiotensin 1-7 plays a role in peripheral vasodilatation, diuresis, and antiproliferation via NO, kinin and prostaglandin release (Hilchey and Bell-Quilley, 1995[20]). In patients treated with ACE inhibitors, increased angiotensin 1-7 levels are claimed to be related with cognitive function improvement (Tom et al., 2003[30]; Von Bohlen und Halbach and Albrecht, 2006[32]). Although the difference between the ACEi and ARBs is yet unclear, angiotensin 1-7 levels also increase with an ARB treatment (Schindler et al., 2007[29]). 

Among all biochemical parameters, the role of angiotensin IV in RAAS was the least clarified. In the present study, plasma angiotensin IV levels of SMMSE > 26 group were higher than plasma angiotensin IV levels of SMMSE = 26 group but this difference did not reach a statistically significant level. Stimulation of the AT4 receptor had a positive effect on cognitive effects such as memory and learning in animal studies that administered angiotensin IV (Albiston et al., 2004[2]; Braszko, 2004[6]; Lee et al., 2004[25]; Gard, 2008[12]). Although it is too early to come to a conclusion, the cognitive improvement provided by ARBs could be related with angiotensin IV. Animal studies showed angiotensin IV had positive effects on cognitive functions. In the present study, there was no correlation between MMSE scores and angiotensin IV concentrations. A possible explanation for this is that angiotensin-specific receptors are mostly found in the brain and show their effects via the local RAAS. Prospective trials are needed to document the effect of angiotensin IV on cognitive processes.

One of the remarkable results in our study was that there was no correlation between blood pressure and angiotensin 1-7 or angiotensin II, but angiotensin IV levels were inversely correlated with both systolic and diastolic blood pressure (p < 0.005). Angiotensin IV is mostly localized in the brain and interacts with AT1 and AT2 receptors as well as AT4 (Capponi and Catt, 1979[8]; Handa et al., 1999[19]; Le et al., 2002[24]). In a rat trial, angiotensin IV administered as i.v. bolus increased arterial blood pressure, this effect was prevented by AT1 blockage (Yang et al., 2008[35]). Angiotensin IV might stimulate AT1 receptors in the brain and increase sympathetic outflow and blood pressure (Lochard et al., 2004[26]). Some effects caused by angiotensin IV are believed to be through AT1 receptor stimulation (Fitzgerald et al., 1999[11]; Gardiner et al., 1993[13]; Yang et al., 2008[35]). In the present study, the negative correlation between angiotensin IV and blood pressure was in contrast to this information; however, it should be kept in mind that 33 of our cases were treated with at least one ARB. In this condition, angiotensin IV might not show the effects via AT1 receptor. In other respects, Spearman’s rho correlation analysis showed a strong linear correlation between angiotensin II and IV (r = 0.766, p < 0.001), angiotensin II and angiotensin 1-7 (r = 0.848, p < 0.001), and angiotensin 1-7 and angiotensin IV (r = 0.745, p < 0.001). Considering that angiotensin 1-7 and angiotensin IV are mid-products of RAAS, these correlations are expected result. In this sense, negative correlation between angiotensin IV and systolic blood pressure might also be explained as angiotensin II’s and angiotensin IV’s effects via AT2 receptor and vasodilator effect of angiotensin 1-7. 

## Conclusions

Compatible with previous findings, independent of antihypertensive-type medications, keeping blood pressure under control to prevent cognitive impairment is one of the important results of the present study. 

Although the present study has a small sample size for arriving at a definite conclusion, our research may support such a hypothesis that system needs to work and increase in angiotensin peptides levels may be associated with better cognitive functions. In other respects, with the exception of the negative effects of angiotensin II on AT1 receptors and high plasma aldosterone levels, the latest findings in this field support that RAAS products and mid-products may have some additional beneficial effects on cognition. ARBs are the only antihypertensive drug group that increase all angiotensin peptides and block AT1 receptors. For this reason, ARBs may provide additional benefit in cognition due to their effects on RAAS. The present study supports this finding. In our study it was not possible to compare ARBs and other antihypertensive drugs because all of the patients in our study were using very different medications. Further larger prospective studies are needed to understand and prove the relation of RAAS biochemical parameters with cognitive function. Future studies should be conducted to evaluate the same parameters pre and post treatment.

There are no guidelines for the choice of antihypertensive drugs for patients with hypertension and cognitive impairment, or to prevent cognitive impairment in patients with hypertension. Antihypertensive drugs target several points on the RAAS pathway, thus they increase or decrease different metabolites. As shown in Table 1(Tab. 1), four RAAS parameters that might be related with cognitive function are affected differently by antihypertensive drugs. In addition to intensive management of blood pressure, it is believed that if the relation between RAAS parameters and cognitive functions is clarified, there may be an opportunity for selecting antihypertensive drugs or deciding antihypertensive treatment strategies. For the time being, further larger prospective studies are still needed to elucidate the relation of RAAS biochemical parameters with cognitive functions. 

### Limitations

Although we were very careful and sensitive to follow instructions for storage and cold chain, angiotensin levels were measured in blood collected in pre-chilled tubes with EDTA (as a proteinase inhibitor) that contained no other proteinase inhibitors and plasma samples were not extracted, which may have limited our study because it may have decreased peptide degradation. We used tubes with EDTA and performed very strict cold chain to keep the peptide degradation to a minimum.

The present study has a small sample size. Nevertheless; our study can be considered as a pilot study and we found some significant findings that may be useful for further larger studies. 

## Acknowledgements

This work was supported by Scientific Research Projects Coordination Unit of Istanbul University (Project number: 27104).

This study was presented as a poster presentation at the 20^th^ Annual Scientific Meeting of the International Society of Cardiovascular Pharmacotherapy (ISCP), 25-26 June 2015, Buenos Aires, Argentina. 

## Conflict of interest

The authors declare that they have no conflict of interest. 

## Supplementary Material

Supplementary data

## Figures and Tables

**Table 1:**
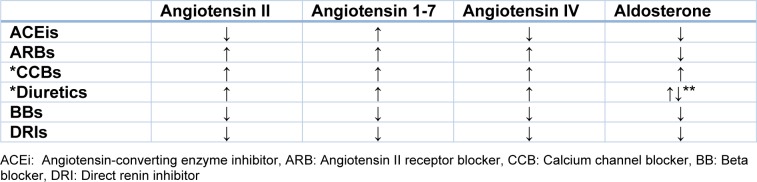
Potential effects of antihypertensive drugs on RAAS parameters. *Although CCBs and Diuretics do not have direct effects on the RAAS system, they possibly activate the RAAS system within compensator mechanisms. ** Potassium-sparing diuretics antagonize effect of aldosterone.

**Table 2:**
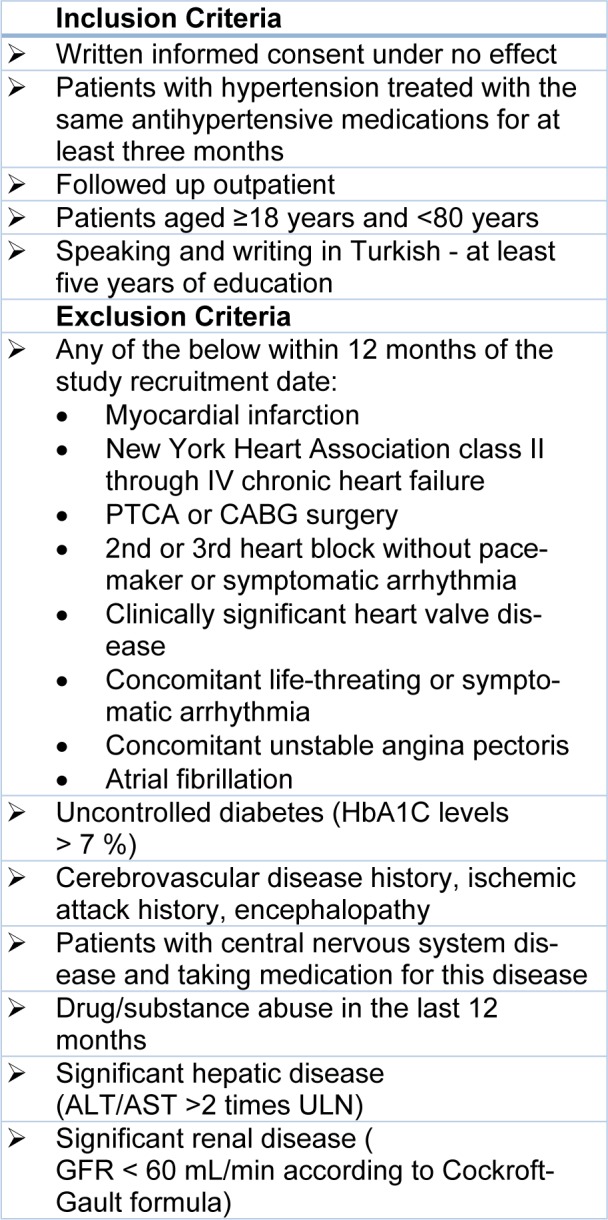
Inclusion and Exclusion Criteria

**Table 3:**
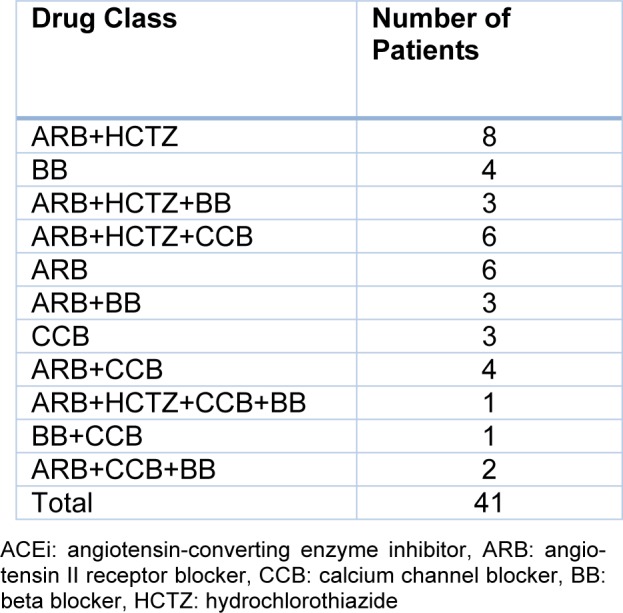
The Patients' Antihypertensive Medications

**Table 4:**
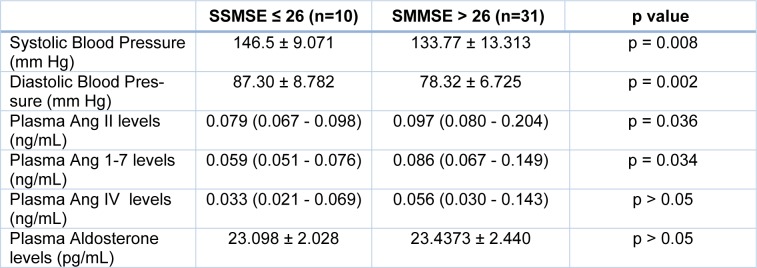
The differences between the groups according to SMMSE scores. The results are presented as mean ± standard deviation or median (25^th^ -75^th^ percentiles) according to the statistical test.

**Table 5:**
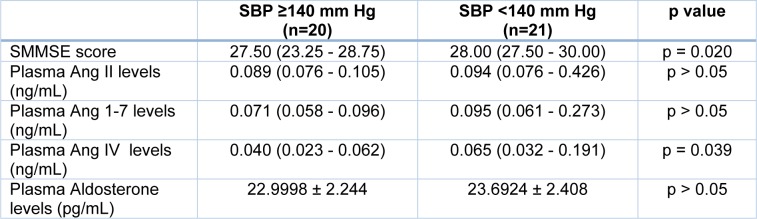
The differences between the groups according to SBP levels. The results are presented as mean ± standard deviation or median (25^th^ -75^th^ percentiles) according to statistical test. SBP: Systolic blood pressure

**Table 6:**
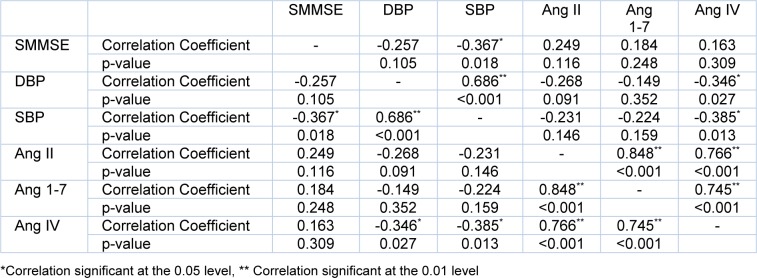
Correlations found in the study. Ang: angiotensin SBP: systolic blood pressure, DBP: diastolic blood pressure
