# Supplementary material for: Evaluating the relationship of blood pressure, plasma angiotensin peptides and aldosterone with cognitive functions in patients with hypertension
Source: EXCLI J. 2017 Mar 10;16:245–55. doi: 10.17179/excli2016-725 (PMC5427471; doi:10.17179/excli2016-725)
Supplement: Supplementary data [file EXCLI-16-245-s-001.pdf]

EXCLI Journal 2017;16:245-255 – ISSN 1611-2156
Received: October 21, 2016, accepted: February 10, 2017, March 10, 2017

Supplementary data to:

Original article:

EVALUATING THE RELATIONSHIP OF BLOOD PRESSURE, PLASMA ANGIOTENSIN PEPTIDES AND ALDOSTERONE WITH COGNITIVE FUNCTIONS IN PATIENTS WITH HYPERTENSION

Selçuk Şen[1*], Nermin Gürel[1], Baran Ufuktepe[1], Zeynep Güneş Özünal[1], Çağla Büyüklü[2], Yağız Üresin[1]

[1] Department of Medical Pharmacology, Istanbul Faculty of Medicine, Istanbul University, 34390 Fatih, Istanbul, Turkey
[2] Department of Neuroscience, Institute of Experimental Medicine, Istanbul University, 34393 Fatih, Istanbul, Turkey

* Corresponding author: Selçuk Şen, Department of Medical Pharmacology, Istanbul Faculty of Medicine, Istanbul University, 34390 Fatih, Istanbul, Turkey;
E-mail: slcsen@istanbul.edu.tr; Phone: +90 212 414 22 40

http://dx.doi.org/10.17179/excli2016-725

This is an Open Access article distributed under the terms of the Creative Commons Attribution License (http://creativecommons.org/licenses/by/4.0/).

http://dx.doi.org/10.17179/excli2016-725

Patient No	Gender	Age(years)	SBP(mmHg)	DBP(mmHg)	sMMSE score	Plasma Ang II (ng/ml)	Plasma Ang 1-7 (ng/ml)	Plasma Ang IV (ng/ml)	Plasma Aldosterone(pg/ml)	Years after First Diagnosis of HT	Medications	sMMSE group	SBP group
**1**	F	69	158	82	28	0,105951	0,086383	0,069821	21,577	5	ARB + HCTZ	1	1
**2**	F	43	128	74	28	0,082621	0,072935	0,064717	23,935	2	BB	1	2
**3**	M	61	133	85	30	0,077454	0,07607	0,049842	24,547	7	ARB + HCTZ + BB	1	2
**4**	M	50	132	79	30	0,635336	0,460691	0,378189	25,946	15	ARB + HCTZ + CCB	1	2
**5**	F	49	146	80	27	0,074594	0,067456	0,062319	21,499	2	BB	1	1
**6**	F	51	122	72	28	0,421882	0,270959	0,220892	24,413	0,4	ARB	1	2
**7**	M	69	121	74	28	0,066026	0,067293	0,055904	23,701	6	ARB + BB	1	2
**8**	M	56	128	84	23	0,07904	0,068962	0,070184	19,949	2	ARB	2	2
**9**	F	66	142	74	28	0,07974	0,07277	0,046953	20,442	10	ARB + HCTZ + CCB	1	1
**10**	F	50	160	93	26	0,078706	0,045922	0,033384	22,581	7	CCB	2	1
**11**	F	58	110	65	27	0,204311	0,149422	0,106656	24,937	5	ARB + HCTZ	1	2
**12**	M	49	129	80	28	0,143432	0,102405	0,041307	24,158	1	ARB + CCB	1	2
**13**	F	69	143	79	29	0,096509	0,0647	0,024329	26,986	5	BB	1	1
**14**	F	62	105	72	29	0,519766	0,335874	0,160669	28,044	13	ARB + HCTZ + BB	1	2
**15**	F	63	149	75	23	0,10834	0,076594	0,034561	22,825	19	ARB	2	1
**16**	F	59	144	75	30	0,101973	0,074616	0,04609	23,721	15	ARB	1	1
**17**	F	45	121	70	30	0,430893	0,275414	0,256957	26,446	10	ARB + BB	1	2
**18**	F	62	155	97	23	0,069232	0,045262	0,017412	26,74	15	ARB + HCTZ	2	1
**19**	F	52	152	88	22	0,073502	0,062391	0,022308	24,025	2	ARB + HCTZ	2	1
**20**	F	52	138	84	26	0,059429	0,052378	0,011084	21,725	2	ARB + HCTZ + CCB	2	2
**21**	M	49	149	93	28	0,069422	0,068233	0,004887	22,466	1,5	ARB + HCTZ	1	1
**22**	M	60	138	84	30	0,582959	0,446966	0,259	24,347	5	CCB	1	2
**23**	M	55	149	88	27	0,080415	0,098949	0,010789	23,188	20	ARB + CCB	1	1
**24**	F	53	145	92	29	0,087206	0,099431	0,014047	21,97	7	ARB + CCB	1	1
**25**	F	61	105	70	27	0,551827	0,594486	0,239806	19,084	18	ARB + HCTZ	1	2
**26**	M	43	136	81	29	0,09164	0,095176	0,049108	22,747	0,5	ARB	1	2
**27**	F	62	150	94	22	0,198881	0,224134	0,078834	22,826	20	ARB + HCTZ + CCB	2	1
**28**	M	65	123	68	28	0,073965	0,040246	0,020275	23,91	15	ARB + HCTZ + CCB + BB	1	2
**29**	F	50	141	78	29	0,098819	0,068788	0,058622	27,44	2	ARB + CCB	1	1
**30**	F	66	146	75	28	0,314221	0,297586	0,241864	20,021	20	ARB + HCTZ + BB	1	1
**31**	F	65	129	72	29	0,094326	0,059149	0,034671	23,088	15	BB	1	2
**32**	F	63	154	89	29	0,132192	0,103668	0,059213	21,596	5	BB + CCB	1	1
**33**	M	67	122	80	30	0,068305	0,062278	0,028639	28,922	20	CCB	1	2
**34**	M	53	145	100	24	0,095008	0,075568	0,068963	21,734	2	ARB + HCTZ + CCB	2	1
**35**	M	65	132	80	27	0,137998	0,119973	0,073992	22,299	1	ARB + BB	1	2
**36**	F	62	146	84	22	0,079919	0,055879	0,027482	22,558	15	ARB + HCTZ + CCB	2	1
**37**	F	68	141	79	28	0,090298	0,056119	0,049966	19,783	15	ARB + HCTZ	1	1
**38**	F	55	138	77	29	0,060305	0,042351	0,023134	21,629	20	ARB + CCB + BB	1	2
**39**	M	47	132	82	30	0,089272	0,040453	0,029571	21,543	5	ARB + CCB + BB	1	2
**40**	F	49	142	74	26	0,057992	0,05201	0,033343	26,018	3	ARB	2	1
**41**	M	61	133	79	28	0,117933	0,099403	0,143166	22,17	13	ARB + HCTZ	1	2

Abbreviations
SBP: systolic blood pressure
DBP: diastolic blood pressure
Ang: Angiotensin
ARB: Angiotensin II receptor blocker
CCB: Calcium channel blocker
BB: Beta blocker
HCTZ: hydrochlorothiazide
sMMSE: Standardized Mini Mental State Examination
HT: Hypertension

Code İdentifiers
sMMSE group: 1 (sMMSE score>26), 2 (sMMSE score ≤26)
SBP Group: 1 (SBP ≥140 mmHg), 2 (SBP < 140 mm Hg)
Gender: F (Female), M (Male)
